# The effects of dislocations on crystallographic twins and domain wall motion in magnetite at the Verwey transition

**DOI:** 10.1186/s40623-018-0981-7

**Published:** 2019-01-15

**Authors:** Anna K. Lindquist, Joshua M. Feinberg, Richard J. Harrison, James C. Loudon, Andrew J. Newell

**Affiliations:** 1 Geology Department, Macalester College, 1600 Grand Ave, Saint Paul, MN 55105 USA; 2 Department of Earth Sciences, Institute for Rock Magnetism, University of Minnesota, 116 Church Street SE, Minneapolis, MN 55455 USA; 3 Department of Earth Sciences, University of Cambridge, Downing Street, Cambridge, CB2 3EQ UK; 4 Department of Materials Science and Metallurgy, University of Cambridge, 27 Charles Babbage Road, Cambridge, CB3 0FS UK; 5 Marine, Earth, and Atmospheric Sciences, North Carolina State University at Raleigh, 2800 Faucette Drive, Raleigh, NC 27695 USA

**Keywords:** Verwey transition, Magnetite, Dislocations, Domain wall, Transmission electron microscopy, TEM, Low-temperature demagnetization, Oxidation, Phase transition

## Abstract

Pure magnetite experiences a first-order phase transition (the Verwey transition) near 120–125 K wherein the mineral’s symmetry changes from cubic to monoclinic. This transformation results in the formation of fine-scale crystallographic twins and is accompanied by a profound change in magnetic properties. The Verwey transition is critical to a variety of applications in environmental magnetism and paleomagnetism because its expression is diagnostic for the presence of stoichiometric (or nearly stoichiometric) magnetite and cycling through the Verwey transition tends to remove the majority of multidomain magnetic remanence. Internal and external stresses demonstrably affect the onset of the Verwey transition. Dislocations create localized internal stress fields and have been cited as a possible source of an altered Verwey transition in deformed samples. To further investigate this behavior, a laboratory-deformed magnetite sample was examined inside a transmission electron microscope as it was cooled through the Verwey transition. Operating the microscope in the Fresnel mode of Lorentz microscopy enabled imaging of the interactions between dislocations, magnetic domain walls, and low-temperature crystallographic twin formation during the phase transition. To relate the observed changes to more readily measurable bulk sample magnetic behavior, low-temperature magnetic measurements were also taken using SQUID magnetometry. This study allows us, for the first time, to observe the Verwey transition in a defect-rich area. Dislocations, and their associated stress fields, impede the development of monoclinic magnetite twin structures during the phase transition and increase the remanence of a magnetite sample after cooling and warming through the Verwey transition.

**Electronic supplementary material:**

The online version of this article (10.1186/s40623-018-0981-7) contains supplementary material, which is available to authorized users.

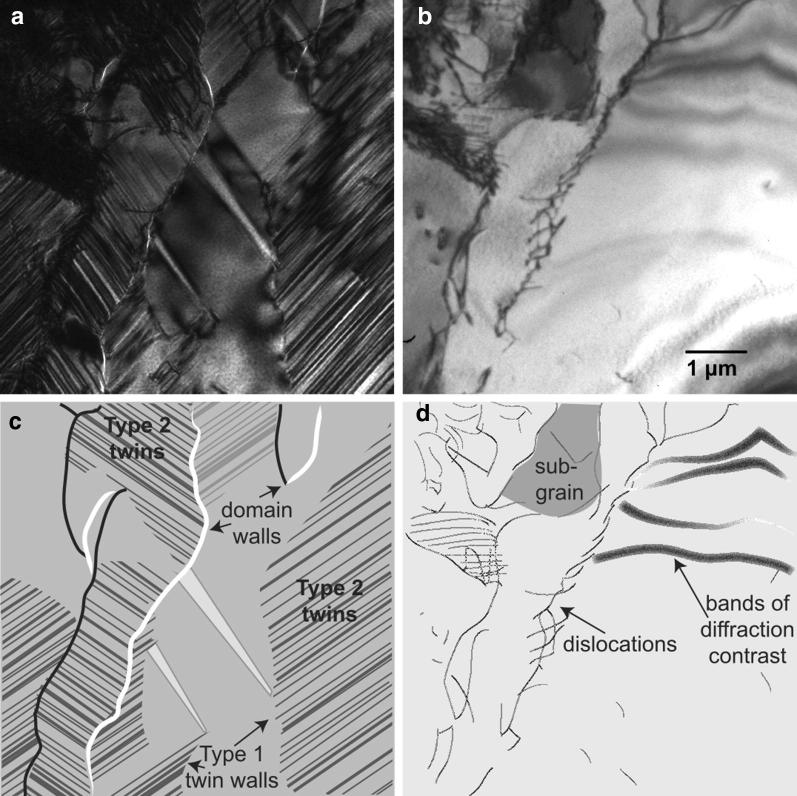

## Introduction

Magnetite is ubiquitous in nature and has broad applications, including in Earth science (e.g., Lascu and Feinberg 2011; Geiss et al. 2008), solid-state physics (e.g., Goya et al. 2003), and biology (e.g., Kirschvink and Gould 1981). At room temperature, magnetite ($\hbox {Fe}^{3+}_{2}\hbox {Fe}^{2+}\hbox {O}^{2-}_{4}$) has an inverse spinel structure in which the oxygen ions are arranged in cubic close-packed (ccp) structure. Half of the $\hbox {Fe}^{3+}$ ions are tetrahedrally coordinated, and the remaining $\hbox {Fe}^{3+}$ ions and the $\hbox {Fe}^{2+}$ ions are octahedrally coordinated.

At $\sim$ 120 K, magnetite undergoes a first-order phase transition, called the Verwey transition (Li 1932; Verwey and Haayman 1941). At the Verwey temperature ($T_{\mathrm{v}}$), the crystallographic structure of magnetite switches from cubic ($Fd{\bar{3}}m$) to monoclinic (*Cc*) (Kasama et al. 2013; Iizumi et al. 1982; Senn et al. 2012). This transformation is accomplished via slight, predictable, and reversible shifts in atomic positions resulting in redefined crystallographic axes such that the new monoclinic *a*, *b*, and *c*-axes correspond to the cubic [1 $\overline{1}$ 0], [110], and [001] axes, respectively (Abe et al. 1976; Iida 1980). The magnetic easy axis changes from the cubic $\langle 111 \rangle$ directions to this new monoclinic [001] (*c*-axis), and the magnetocrystalline anisotropy is 15 times greater (Abe et al. 1976).

### Crystallographic twins

The instantaneous strain generated by the phase transition causes fine-scale crystallographic twins to form within monoclinic magnetite below $T_{\mathrm{v}}$. Three primary twin types form upon cooling; they are described in detail by Kasama et al. (2010, 2013) and Bryson et al. (2013), and briefly summarized as follows.

Type 1 ferroelastic twin walls are coincident with magnetic domain walls, and the magnetic easy axes (*c*-axes) on either side of the twin wall are orthogonal to each other. Type 1 twins form where two separately nucleated regions of monoclinic magnetite converge, forming jagged, uneven boundaries. Type 1 twin walls often separate regions filled with type 2 twins. The latter are defined by a 180$^\circ$ rotation about the *c*-axis, so their magnetic easy, medium, and hard axes are aligned. Type 2 twins are very fine (tens of nm). Unlike types 1 and 3, domain walls move easily through type 2 twins—usually normal to the twin walls. Finally, type 3 twins are typically elongated in shape with straight twin walls. These are characterized by a 90$^\circ$ rotation about this *c*-axis, so the a- and b-axes (the magnetic hard and intermediate axes, respectively) are interchanged. As with type 1 twins, the change in magnetic anisotropy means these crystallographic twin walls often align with magnetic domain walls.

### Magnetic and stress effects on $T_{\mathrm{v}}$

Strong magnetocrystalline anisotropy in the low-temperature magnetite phase allows ambient magnetic fields to influence the selection and preferred orientation of the monoclinic magnetic easy axis. It also allows both internal and external stresses to affect selection of the monoclinic axes. Internal and external stresses can alter both $T_{\mathrm{v}}$ (Liu et al. 2008; Coe et al. 2012; Hodych et al. 1998; Liu et al. 2017) and the remanence held by magnetite after cooling through $T_{\mathrm{v}}$ (Özdemir and Dunlop 1999; Özdemir 2000; Heider et al. 1992; King and Williams 2000). It is believed—though it has not been demonstrated previously—that dislocations play a significant role in these behaviors (e.g., Coe et al. 2012; Muxworthy and Williams 1999; Muxworthy and McClelland 2000).

If the Verwey transition occurs in a field-free environment, any one of the cubic $\langle 100 \rangle$ axes may become the monoclinic [001] axis (Özdemir and Dunlop 1999; Calhoun 1954; Bickford 1953, 1950; Domenicali 1950). However, if the Verwey transition occurs in the presence of a magnetic field, the cubic $\langle 1 0 0 \rangle$ axis most closely aligned with the applied field is energetically favored (Li 1932; Verwey et al. 1947; Williams et al. 1953; Calhoun 1954).

As with applied magnetic fields, physical stress can affect axis selection. Upon conversion to its monoclinic form, the dimensions of a magnetite crystal change slightly such that there is an elongation of 0.31% along an axis near a cubic [1 1 1] axis and shortening within the plane normal to this axis (Coe et al. 2012). The application of non-hydrostatic stress during cooling through the Verwey transition influences the selection of this axis of elongation. $T_{\mathrm{v}}$ is higher for the crystallographic direction experiencing the lowest compressive stress (or the greatest tensile stress) (Coe et al. 2012). Therefore, upon cooling, this crystallographic orientation is thermodynamically favored and will dominate. The shape change associated with the phase transition can also redistribute local stresses, which also influences axis selection through the Verwey transition.

Internal stresses can also modify the expression of the Verwey transition. For example, stresses arising from crystallographic defects and non-stoichiometry have been shown to broaden and lower the Verwey transition (Hodych et al. 1998). They have also been shown to increase remanence upon cooling below the Verwey transition (Heider et al. 1992; King and Williams 2000).

Low-temperature demagnetization (LTD) has long been used as an important means for removing unwanted multidomain remanence in paleomagnetic specimens (Ozima et al. 1964; Merrill 1970; Schmidt 1993). By cooling a specimen through the Verwey transition and then warming it back to room temperature, it is possible to remove most of the remanence held by multidomain grains, leaving only the stable single-domain and harder pseudo-single-domain components of magnetic remanence. During LTD, loosely pinned domain walls are freed as the magnetite passes through its isotropic point ($T_{\mathrm{i}} \approx 130$ K), the temperature at which magnetic anisotropy reaches zero (Özdemir 2000). The component of magnetization that remains after low-temperature demagnetization (LTD) is stable enough to withstand the crystallographic shifts that occur during the Verwey transition (Özdemir and Dunlop 1999). Heider et al. (1992) found that higher internal stress, even within larger multidomain magnetite grains, enabled a sample to better retain a stable memory of magnetic remanence. Hodych et al. (1998); Özdemir and Dunlop (1999), and Özdemir (2000) proposed that magnetic domain walls may pin strongly enough at some crystallographic defects to persist through LTD.

For decades, published studies reported $T_{\mathrm{v}}$ decreased as increasing pressures were applied (e.g., Kakudate et al. 1979; Rozenberg et al. 1996; Todo et al. 2001). More recent studies have reported that internal stresses can increase $T_{\mathrm{v}}$ (e.g., Carporzen and Gilder 2010; Bezaeva et al. 2016; Kontny et al. 2018). Computational work by Coe et al. (2012) found that hydrostatic stress depresses $T_{\mathrm{v}}$, but that uniaxial stresses less than $\sim 0.8$ GPa increase $T_{\mathrm{v}}$ (assuming the low-temperature magnetite grain is free to form twins with any of the 12 possible crystallographic orientations). The relationship between stress and $T_{\mathrm{v}}$ is further complicated by physical barriers (e.g., crystalline imperfections and inclusions) and magnetic fields because these constrain twin selection. Coe et al. (2012) suggest broadened Verwey transition temperatures may be the result of complex internal stress fields—often the result of dislocations and other defects—causing variable localized changes in $T_{\mathrm{v}}$.

### This study

This study examines the role of dislocations and dislocation networks on the Verwey transition. Dislocations are linear crystallographic defects within the crystal structure of a material that create local stress fields. Theory (Stacey and Wise 1967; Xu and Merrill 1989; Moskowitz 1993) and direct observational experiments (Lindquist et al. 2015) have shown that dislocations can pin domain walls and increase coercivity in cubic multidomain magnetite, but very little is known about the effects of these defects on the Verwey transition and the monoclinic form of magnetite. Earlier studies (Heider et al. 1987; Liu et al. 2008) suggest that dislocations may affect the Verwey transition.

This study directly images the interactions between dislocations, crystallographic twin formation, and magnetic domain structure through the Verwey transition. It combines Lorentz mode transmission electron microscopy (TEM) with low-temperature magnetic measurements in an effort to better understand the impact of dislocations on the Verwey transition and to extrapolate these micrometer-scale behaviors to more readily measurable bulk magnetic properties.

## Methods

### Sample preparation and description

The samples used for this study were cut from an octahedron of natural magnetite and deformed uniaxially in a mixed $\hbox {CO/CO}_{2}$ environment using a one-atmosphere rig in the Rock Deformation Lab at the University of Minnesota. The goal of these experiments was to create dislocations and other deformation microstructures in a highly controlled environment. The deformation conditions are summarized in Table 1. A more complete description of the deformation experiments and the interactions between the resultant dislocations and magnetic domain walls at room temperature can be found in Lindquist et al. (2015).

**Table 1:** Summary of deformation conditions from Lindquist et al. (2015), with resulting strain rate, dislocation density, and hysteresis parameters. DS0 is the undeformed sample. *T* is temperature at which deformation occurred, *P* is applied pressure during deformation, t is duration of deformation experiment, ${\dot{\epsilon }}$ is strain rate, $\rho$ is dislocation density as measured from etched samples imaged in the SEM, $H_{\mathrm{c}}$ is coercivity, $M_{\mathrm{r}}$ is magnetic remanence, and $M_{\mathrm{s}}$ is the saturation magnetization

	*T* ($^{\circ }$C)	*P* (MPa)	*t* (h)	${\dot{\epsilon }}$ ($10^{-9} 1/\hbox {s}$)	$\rho$ ($10^{10} 1/\hbox {m}^{2}$)	$H_{\mathrm{c}}$ (mT)	$M_{\mathrm{r}}$ ($\hbox {Am}^{2}/\hbox {kg}$)	$M_{\mathrm{s}}$ ($\hbox {Am}^{2}/\hbox {kg}$)
DS0	n/a	n/a	n/a	n/a	0.84 ± 0.16	0.29	0.157	93.5
DS1	1011	50.6	24	$91\pm 2$	1.3 ± 0.56	0.36	0.334	92.7
DS2	853	38	67	$3.4\pm 3.0$	11 ± 6.2	0.53	0.466	103
DS3	989	19.8	69	$2.6\pm 1.8$	4.2 ± 2.5	0.36	0.427	91.5
DS4	705	50.3	120	$1.3\pm 1.3$	21 ± 10	0.37	0.189	92.8

Sample DS4 is the primary sample of interest for this paper and was the only sample used for TEM imaging. DS4 contains a heterogeneous distribution of complex dislocation structures, defect-free areas, and regions with isolated dislocations. In preparation for TEM imaging, $\sim$ 2-mm slices were cut from a rectangular rod of magnetite. These slices were then wedge-polished following the steps outlined in Voyles et al. (2003), with the only notable difference being the use of ion milling as a final step instead of a polishing cloth and colloidal silica.

### TEM imaging

TEM imaging was carried out at the University of Cambridge in the Department of Materials Science and Metallurgy using a Philips CM300 TEM with an accelerating voltage of 300 kV and a Gatan sample stage that was capable of being cooled with liquid nitrogen or warmed using a small heater. Using this holder, the sample was cooled to approximately 90 K. The sample was cooled and warmed multiple times using the sample stage, making it possible to repeatedly watch the transition through the Verwey temperature.

The imaging for this study was conducted in a Fresnel Lorentz mode wherein the objective lens was turned off and the diffraction lens used to focus the image. By operating the microscope in Lorentz mode (for more information on Lorentz mode electron microscopy, see Chapman 1984), it was possible to image both magnetic domain walls and crystallographic twins simultaneously. Despite not using the objective lens, a small vertical field of 16.8 mT remained in the vicinity of the sample during imaging. The sample was nominally aligned so that the [112] axis was parallel to this field.

### Low-temperature magnetic measurements

Zero-field-cooled (ZFC) and field-cooled (FC) remanence and room temperature saturation isothermal remanent magnetization (RTSIRM) on cooling and warming were measured at the Institute for Rock Magnetism on the Quantum Designs Magnetic Properties Measurement Systems (MPMS). For ZFC measurements, the sample was cooled to 10 K in a zero-field environment, and a 2.5 T isothermal remanent magnetization (IRM) was imparted. Measurements were taken every 5 K as the sample warmed from 10 to 300 K in a field-free environment. FC measurements were taken in the same manner, except the sample was initially cooled in a 2.5 T field, which was turned off once the sample was cooled to 10 K. RTSIRM were measured after applying and removing a 2.5 T field at 300 K. Then, measurements were taken every 5 K as the sample was cooled to 10 K and warmed back to 300 K. RTSIRM measurements are especially useful for observing the magnitude of remanence remaining after low-temperature demagnetization.

## Results

The videos demonstrating the interactions between dislocations and twin formation are available in the supplemental materials. Please note that in both videos, the temperature indicated is approximate because the temperature of the sample holder is not measured immediately adjacent to the sample, which may experience localized heating due to interactions with the electron beam. The temperature has been included as a reference to indicate cooling and warming and is not intended to be an absolute measure of $T_{\mathrm{v}}$. As cooling continues below $T_{\mathrm{v}}$, shifts in twin boundaries are observable (especially in Additional file 2: Video 2), as suggested by Dunlop et al. (2018); Kosterov and Fabian (2008).

**Fig. 1:**
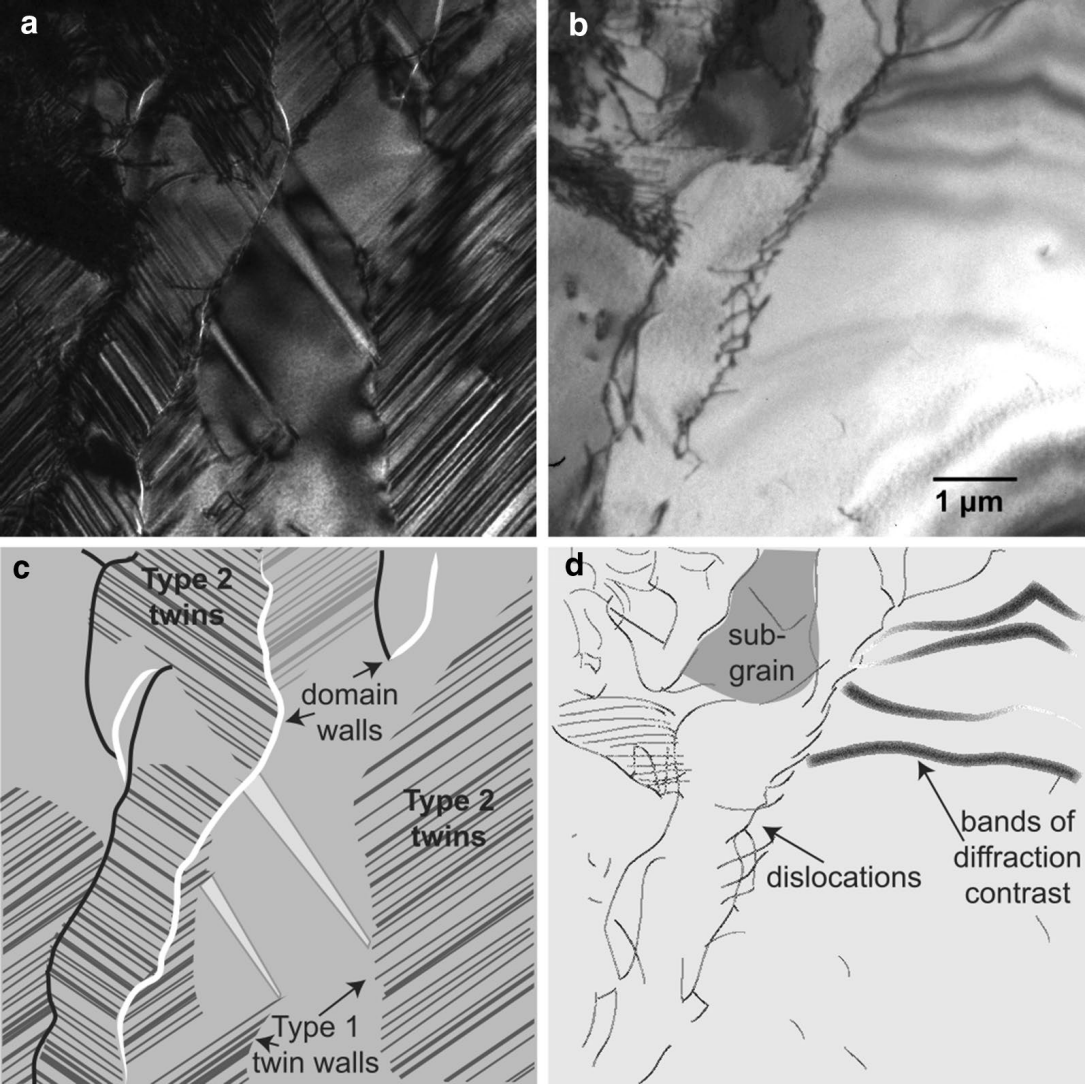
TEM images showing domain walls, dislocations, and twin structures below the Verwey transition. All four images show the same region. **a** Dislocation-rich region below the Verwey transition. **b** Dislocation-rich region above the Verwey transition to illustrate the relationship between twin boundaries and dislocations. **c** Illustration describing image **a**. **d** Illustration describing image **b**

### Twin formation

Videos and images show dislocations affecting twin formation and propagation through the Verwey transition.

The same dislocation-rich region is visible in Fig. 1 and Additional file 1: Video 1, but following different iterations of cooling. Though the twin boundaries do not match exactly, dislocations controlled major twin boundaries following each cooling cycle. The dense dislocation networks consistently impeded twin propagation and were often coincident with the type 1 twin walls, which separate regions filled with type 2 twins. Each observed iteration of cooling through the Verwey transition resulted in type 1 twins dominating in the dislocation-rich region shown in Fig. 1.

**Fig. 2:**
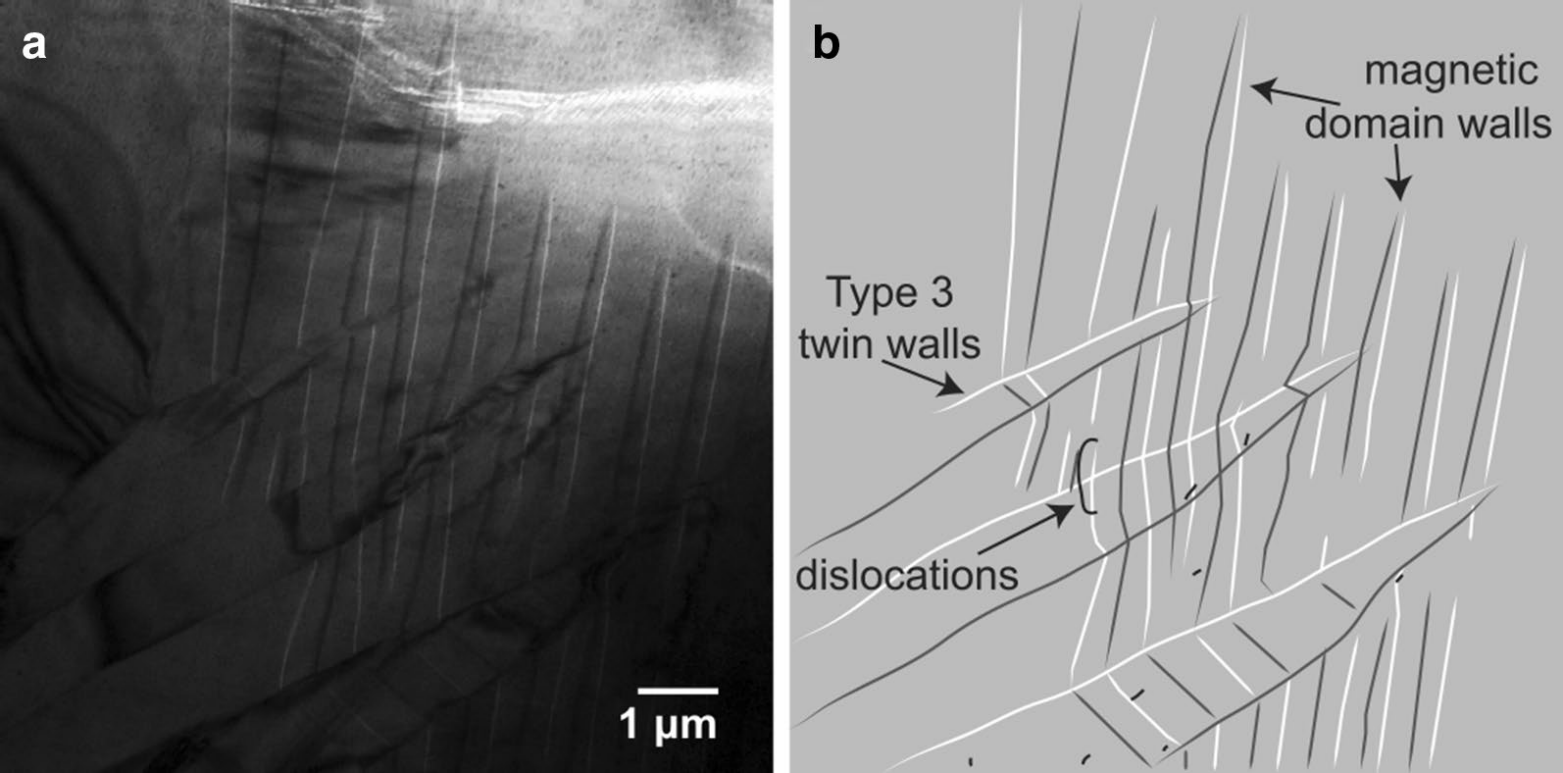
**a** Region with few dislocations below the Verwey transition. **b** Illustration describing image **a**

In Fig. 2, dislocations appear to cause only slight shifts in twin boundaries, and each of the three types of twins were observed during different cooling iterations (Fig. 2 and Additional file 2: Video 2). These more isolated dislocations have variable impacts, but appear to weakly pin both twin walls and the leading edge of the monoclinic phase as it progresses across the sample during cooling. They do not appear to control final twin structures.

### Magnetic measurements

**Fig. 3:**
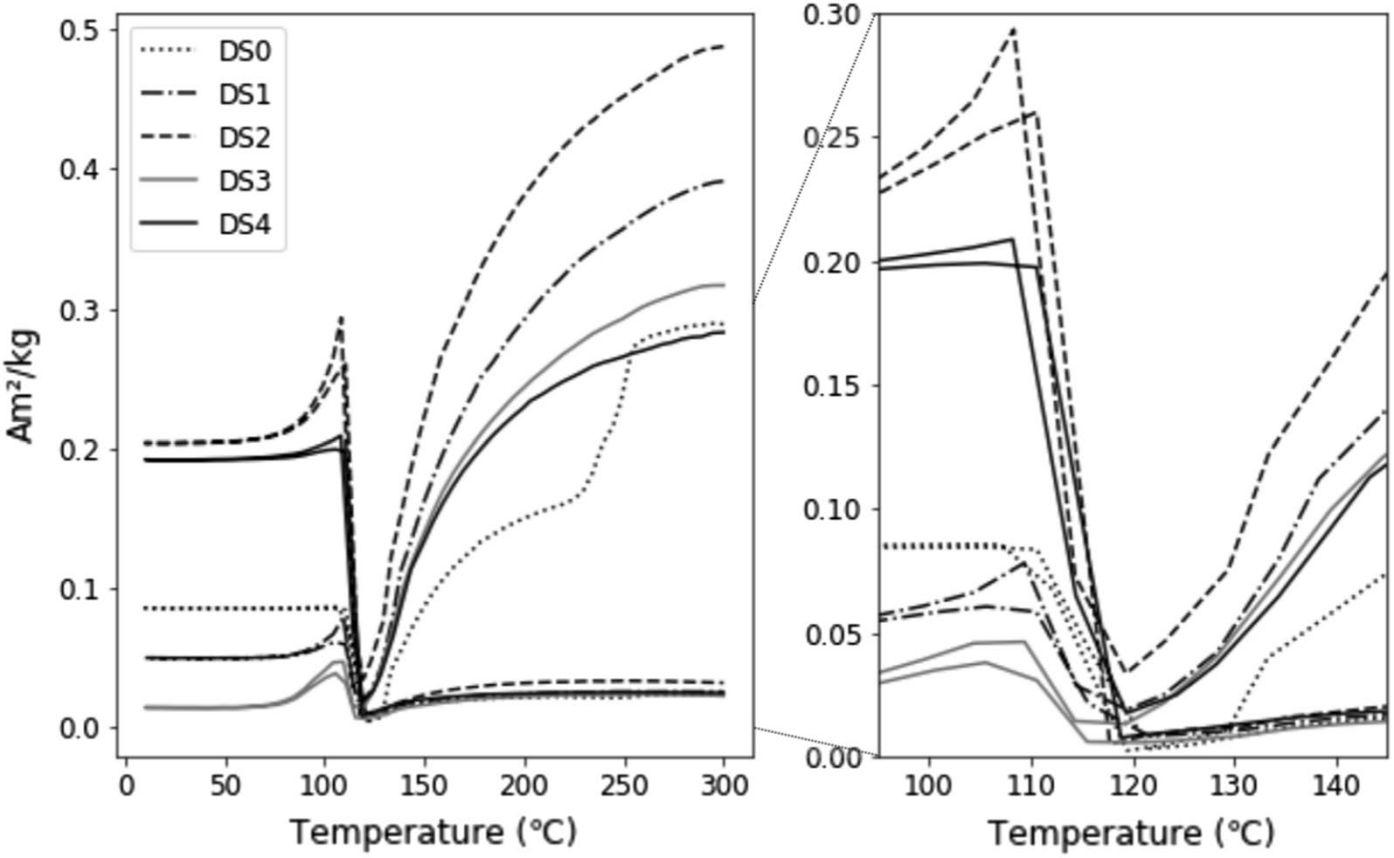
Left shows RTSIRM measurements for all five samples. Samples DS2 and DS4, which were deformed at lower temperatures and have higher dislocation densities, have the highest low-temperature magnetizations. Right shows close-up of behavior around the Verwey transition

RTSIRM experiments (Fig. 3) show steady decrease in magnetization during cooling, reaching a minimum between 115 and 130 K, and then rapid increase to a sharp peak below 115 K, before lowering slightly to reach a magnetization plateau at $\sim$ 75 K. The percentage of remanence lost by the magnetite grains after cycling through the RTSIRM experiment was calculated to be between 91.5% (DS4) and 94.0% (DS1) (Fig. 5).

Below the Verwey transition, the mass-normalized magnetic moment differs significantly between samples (Fig. 3). Samples DS2 and DS4, which were deformed at the lowest temperatures and have the highest dislocation densities, have low-temperature magnetizations that are 50 to 200% greater than the magnetizations of DS3 and DS1, which were deformed at higher temperatures and have lower dislocation densities. Further, their low-temperature magnetization is only $\sim 30\%$ lower than their room temperature magnetization, while the other samples lose $>60\%$ of their initial magnetization upon cooling through $T_{\mathrm{v}}$ and $T_{\mathrm{i}}$.

**Fig. 4:**
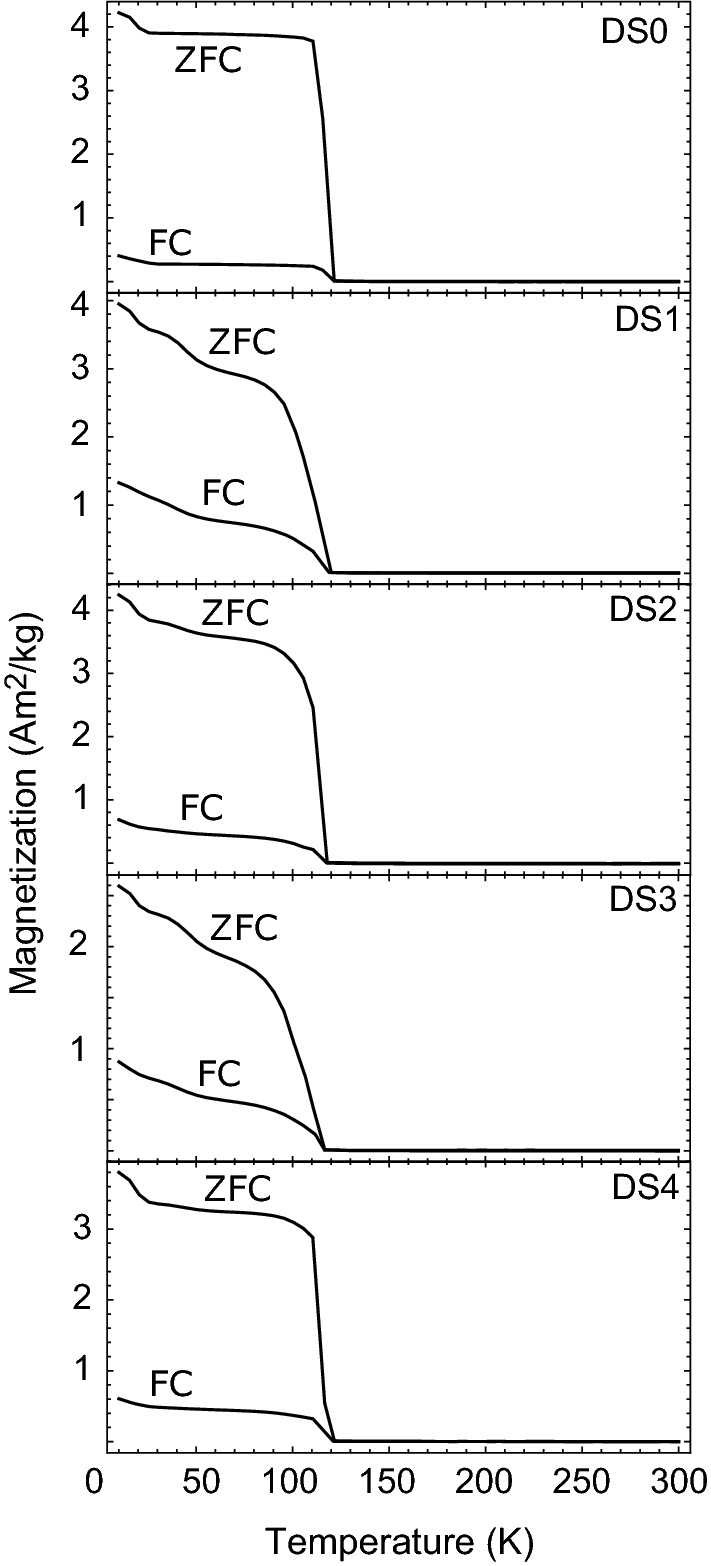
Field-cooled (FC) and zero-field-cooled (ZFC) curves for all five samples

FC and ZFC magnetization measurements (Fig. 4) show the development of a broader, more soft-shouldered Verwey transition in the deformed samples when compared to sample DS0, though all display a clear Verwey transition. This behavior is most pronounced in the samples deformed at higher temperatures (DS1, DS3) and very limited in DS4 (which was deformed at the lowest temperature). In all instances, the ZFC magnetization was significantly larger than the FC magnetization below the Verwey transition. This indicates the 2.5 T biasing field dramatically improves the selection efficiency of the monoclinic [001] during cooling, so fewer twins are present. Domain walls can then move more easily through the sample, resulting in lower net magnetization (Smirnov 2009; Kosterov and Fabian 2008).

For DS0, the undeformed sample, the Verwey transition occurs around 115 K as a very clear and rapid transition. In the remaining, deformed samples, the Verwey transition is a more gradual transition that occurs over an approximately 20 K temperature span. Samples DS1 and DS3, which were deformed at higher temperatures but have lower dislocation densities, display the most gradual Verwey transition behavior. Additionally, these samples displayed slight inflections near 50K in both the FC and ZFC data. Similar inflection points were also observed in low-temperature hysteresis measurements by Dunlop et al. (2018). Samples DS2 and DS4, which had higher dislocation concentrations, and lower strain rates, have relatively abrupt Verwey transitions and no clear inflections near 50 K.

## Discussion

### Dislocation effects on twin formation

Twin formation in regions of relatively low dislocation density (Additional file 2: Video 2 and Kasama et al. 2010) progresses smoothly. In contrast, twin formation in dislocation-rich regions (Additional file 1: Video 1) appears more halting, moving almost in a stick–slip manner. Dislocation stress fields are strongest immediately adjacent to the dislocations (Stacey and Wise 1967; Xu and Merrill 1989; Moskowitz 1993). These likely affect twin propagation by causing small-scale variations in $T_{\mathrm{v}}$. It is also possible that the dislocations, if appropriately aligned, would physically impede twin propagation. Dislocation structures cause additional subdivisions within the sample that must each independently nucleate and develop monoclinic magnetite. Additional file 1: Video 1 and Fig. 1 illustrate such dislocation-bounded twin structures. Additional file 2: Video 2 and Fig. 2, however, demonstrate that smaller more sparsely distributed dislocations are less likely to have the same degree of influence on twin formation and arrangement.

The effects of dislocations on twin formation are likely linked, in part, to the orientation of dislocations and the types of twins. Because dislocations do not uniformly stress the crystal around them, some twins may be unaffected by their neighboring dislocations. This is especially true for type 2 twins, which have equivalent easy, medium, and hard magnetic axes across the twin boundaries. Indeed, fine-scale type 2 twins appear to dominate in dislocation-rich regions (Fig. 1), whereas all three types of twins are found in regions with low dislocation density.

Magnetic domain walls are strongly pinned wherever crystallographic twins and dislocations coincide in the monoclinic magnetite. In classical one dimensional models of domain wall pinning at dislocations in cubic magnetite (e.g., Stacey and Wise 1967; Xu and Merrill 1989; Moskowitz 1993), a domain wall must only overcome the pinning energy associated with a dislocation. (This behavior can be observed in Lindquist et al. 2015). In low-temperature magnetite, a domain wall must overcome the pinning energy of both the dislocation and the crystallographic twin boundary. Because of the high magnetocrystalline anisotropy in low-temperature magnetite, this domain wall movement would likely occur in conjunction with twin wall movement. Thus, high concentrations of dislocations will constrain the development of twin boundaries as well as the magnetic domain structure in low-temperature magnetite (though the twin walls will not always coincide with domain walls). Regions with high dislocation densities would also severely limit domain wall motion.

### Low-temperature demagnetization

**Fig. 5:**
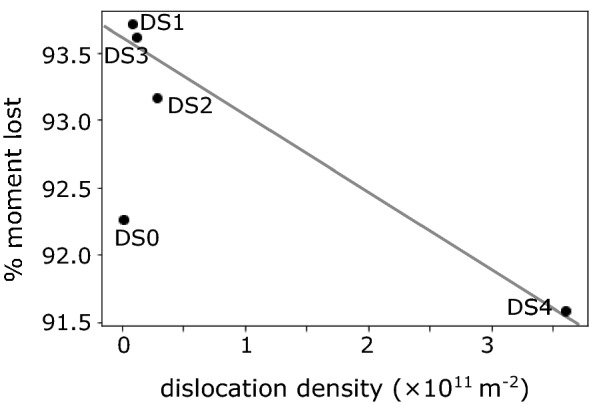
Percent of net magnetic moment lost after cooling through the Verwey transition and warming back to room temperature versus dislocation density. Moment lost calculated from RTSIRM measurements. The trend line is defined by the equation: %moment lost $= -5.6\times 10^{-12} \rho + 94$, where $\rho$ is the dislocation density in 1/m$^2$. DS0 was not included in the trend line calculation

Samples with a higher dislocation density retained a higher remanence after LTD (Fig. 5). This relationship demonstrates the ability of dislocations to increase stable remanence. Liu et al. (2008) demonstrated that for multidomain magnetite, it is the high-coercivity component of magnetization that persists following LTD. Dislocations are then likely to be responsible for increased remanence in samples DS2 and DS4. The drop in remanence in sample DS0 that occurs around 249 K may be indicative of domain reorganization as cubic anisotropy changes upon cooling, but because of this anomalous behavior, the sample was excluded from the regression analysis in Fig. 5.

Dislocation pinning of domain walls through LTD was not captured in our videos. This is likely because the TEM maintained a 16.8 mT field during imaging, which is a sufficiently strong field to allow most domain walls to overcome the pinning coercivity associated with dislocations as the sample was cooled through the isotropic point. Further, the videos and images used for this study focus only on two very small regions within a larger multidomain sample. We interpret our results here to suggest that regions with higher dislocation densities are more likely to pin magnetic domain walls upon cooling through $T_{\mathrm{v}}$.

### Magnetic behavior through $T_{\mathrm{v}}$

RTSIRM measurements show that samples with higher dislocation densities, DS2 and DS4, display dramatic increases in remanence upon cooling through $T_{\mathrm{v}}$. $\Delta _{VJ}$ has been associated with high-coercivity phases in pseudo-single-domain and multidomain magnetite (Muxworthy et al. 2003). In these samples, higher dislocation density and higher coercivity both correspond to higher $\Delta _{VJ}$ values, which follows this reported trend (Table 2). Further, in the deformed samples, remanence peaks are visible immediately below the Verwey transition. A similar peak was observed in (unaltered) single crystals of magnetite in Özdemir and Dunlop (1999). However, our undeformed magnetite does not show this remanence peak; it only appears in the deformed samples.

One notable difference in the FC and ZFC measurements is that the onset of the Verwey transition is more soft-shouldered in some samples. This is most pronounced in samples DS1 and DS3, though it is visible to a lesser degree in DS2. Isothermal annealing at higher oxygen fugacities can increase non-stoichiometry in magnetite, thereby altering $T_{\mathrm{v}}$ (Aragon et al. 1985). Non-stoichiometry (for example additional oxygen or titanium) can suppress the Verwey transition, modulate the temperature at which it occurs, or eliminate it completely (Verwey and Haayman 1941; Verwey et al. 1947; Kakol and Honig 1989a, b; Kakol et al. 1992; Özdemir et al. 1993; Brabers et al. 1998). Because the observed behavior is more pronounced in samples deformed at higher temperatures, it may then be tied to non-stoichiometry. Indeed, it is consistent with studies on partially oxidized magnetite which found higher degrees of non-stoichiometry resulting from oxidation broadened and lowered the Verwey transition temperature (Özdemir et al. 1993; Carporzen and Gilder 2010; King and Williams 2000). It is worth noting that some of this behavior could also be the result of increased mobility of twin boundaries near the transition temperature (Kosterov and Fabian 2008)

**Table 2:** Calculated $\Delta _{VJ}$ values from RTSIRM measurements for each sample, tabulated with dislocation density and coercivity (also in Table 1). $\Delta_{VJ}$ was calculated by subtracting the minimum magnetization at $T_{\mathrm{v}}$ from the maximum magnetization approximately 10 K below $T_{\mathrm{v}}$, as described in Muxworthy et al. (2003)

	$\rho$ ($10^{10}$ 1/m$^2$)	$\Delta _{VJ}$ Am$^2/kg$	$H_{\mathrm{c}}$ (mT)
DS0	0.84 ± 0.16	0.29	0.29
DS1	1.3 ± 0.56	0.15	0.36
DS2	11 ± 6.2	0.53	0.53
DS3	4.2 ± 2.5	0.10	0.36
DS4	21 ± 10	0.68	0.37

### Effects on $T_{\mathrm{v}}$

**Fig. 6:**
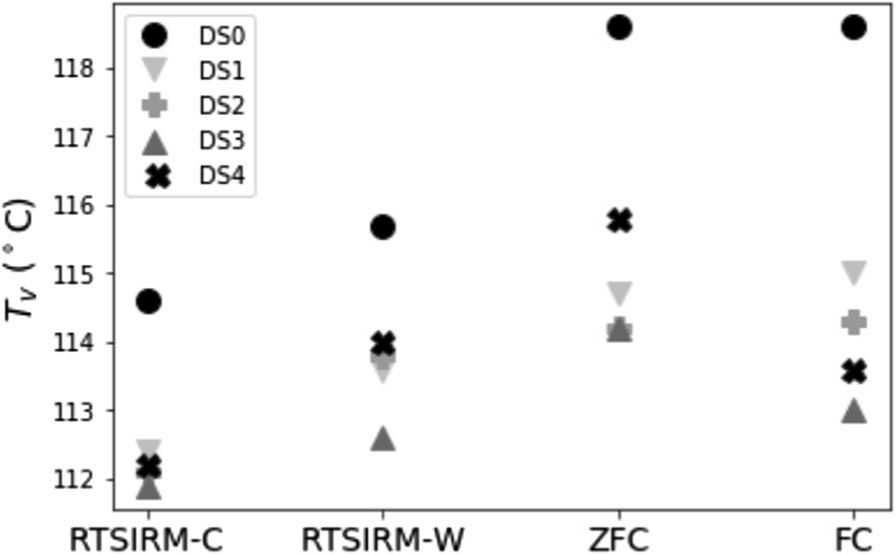
Verwey temperatures from each of the four low-temperature measurements. RTSIRM-C indicates $T_{\mathrm{v}}$ measured during the cooling portion of the RTSIRM measurements, and RTSIRM-W is $T_{\mathrm{v}}$ measured upon warming. Verwey temperatures were determined by assuming the minimum value of the first derivative of the curve represents the Verwey transition. Error bars for each point are ± 2.5$^\circ$ C

Deformation lowered $T_{\mathrm{v}}$ relative to DS0 in all samples (Fig. 6). Coe et al. (2012) found that moderate uniaxial stress raises $T_{\mathrm{v}}$ if all twelve twins are free to form. However, if twin formation is additionally constrained, uniaxial stress can lower $T_{\mathrm{v}}$. In our samples, the complicated dislocation stress fields and sample magnetization appear sufficient to constrain twin selection, depressing $T_{\mathrm{v}}$ in the deformed samples. However, when calculated from the RTSIRM data measured upon cooling, variation in $T_{\mathrm{v}}$ is within the measurement error. There is greater variability in $T_{\mathrm{v}}$ when measured upon warming (as in the warming RTSIRM, ZFC, and FC data, Fig. 6), but $T_{\mathrm{v}}$ for samples DS1-4 remains consistently below that of DS0.

## Conclusions

Dislocations cause variable internal stress fields. Some studies have suggested these stress fields would affect the onset of the Verwey transition (Coe et al. 2012; Kakudate et al. 1979; Liu et al. 2008). TEM videos and images support this assertion, but magnetic measurements suggest that non-stoichiometry is more likely to be responsible for a broadening of the Verwey transition. The combined effects of complex dislocation structures, sample magnetization, applied magnetic fields, and non-stoichiometry slightly depress $T_{\mathrm{v}}$ in our deformed samples.

Samples with higher dislocation density retain a larger magnetization after LTD, supporting the assertion that dislocations can pin domain walls upon cooling and rewarming through $T_{\mathrm{v}}$. These dislocation-rich samples also displayed larger $\Delta _{VJ}$ through the Verwey transition, supporting the assertion that sample microcoercivity is linked to $\Delta _{VJ}$.

Dislocations impede twin formation below the Verwey transition by acting as a barrier to the spread of the monoclinic phase. Even smaller, isolated dislocations (which did not control twin formation) temporarily pinned twin walls and slowed the spread of the monoclinic phase.

As low-temperature monoclinic twins form in magnetite, longer and more complex dislocation structures impede twin propagation through the crystal. These regions with high dislocation density seem to favor the formation of type 1 and 2 twins. Type 1 twins pin along dislocation structures, likely because these twin boundaries are unable to progress through the dislocations as they propagate across the sample. Similarly, magnetic domain walls appear unable to overcome the pinning of these dislocation–twin wall complexes. Type 2 twins form in the space between dense dislocation tangles. This may be because they form on a finer scale than other twin types and have equivalent magnetic easy, medium, and hard axes, making them better suited to dislocation-rich, high-stress regions.

## Additional files

**Additional file 1: Video 1.** The temperature listed in this video is only an approximation and is not intended to measure* T*_*v*_. In Video 1, the formation of twin structures, primarily type 2 twins, occurs amid a series of dislocation structures marked by small yellow lines. (Note that some small dislocations are not marked.) During the video, sudden jumps in image are the result of the Verwey transition elsewhere in the sample. A white magnetic domain wall aligns with a type 1 twin wall at the leading edge of the type 2 twins advancing across the visible area (starting at t = 82 s) from the top right corner as the sample cools. Ultimately, the type 1 twin boundary (visible as a white line indicating a magnetic domain wall) stops at a network of dislocations (t = 98 s). In multiple iterations of cooling and warming, this dislocation network consistently aligns with a type 1 twin boundary (e.g., Fig. 1) and a magnetic domain wall. In the bottom of the sample, the upward development of type 2 twins pins at another dislocation before jumping forward. Black wedge on left of field of view is the sample holder and can be ignored. t = 0 s Horizontal white magnetic domain wall visible at bottom of screen. t = 31 s Domain wall jumps off bottom of field of view. t = 60 s A vertical white domain wall, narrow domain, and black domain wall appear on the right side of the image, leave around t = 67 s. t = 84 s A domain wall in upper right corner is a leading edge to monoclinic phase filled with type 2 twins. Type 2 twins are very narrow, creating the fine contrast stripes in the TEM image. t = 96 s The domain wall/type 1 twin wall continues across the sample until pinning at the rightmost dislocations, t = 95 s Type 2 twins wrap below the dislocations and begin to spread upward toward/through the “E”-shaped dislocation structure. As they propagate through this dislocation structure, “jumps” in the image indicate a sudden unpinning of twin boundary as twins spread. t = 99 s Type 2 twins moving through dislocations reach top of field of view. t = 105 s Reorganization of twin structures visible as sudden jumps in contrast. t = 140 s Type 2 twins visible on left of image. Type 1 twin wall corresponds with long dislocation tangle and white domain wall. Dislocation-bounded regions have different twin orientations.

**Additional file 2: Video 2.** This video shows a region of the sample with few dislocations warming then cooling through the Verwey transition. The shorter, more isolated dislocations pin domain walls and twin walls more weakly than do longer dislocation tangles. This video first shows twins disappearing as the sample is warmed. The sample is then cooled, and new twins are observed to form. The largest dislocation in this region is an arc near the upper left side of the field of view. As the type 3 twins develop below this dislocation, a pair of domain walls from these twins continue upward and pin alongside the dislocation arc (t = 94 s). Later, a type 1 twin wall advances from the right side of the screen and is temporarily pinned by these isolated dislocations. This behavior is most evident beginning at t = 106 s when the twin wall joins a white domain wall in the center of the field of view. Once the twin wall and the magnetic domain wall meet, the domain wall remains pinned to the twin wall as it progresses across the field of view. The type 2 twins sweeping behind the type 1 twin wall appear unaffected by the dislocations. t = 0 s Below the Verwey transition. Vertical domain walls and diagonal type 3 twin walls visible amid scattered dislocations (in yellow). t = 8 s Smooth transition to cubic form as monoclinic twins and domain walls break down and combine. t = 30 s Field of view is now above the Verwey transition temperature. A white domain wall is still visible on the right side of the field of view. t = 52 s Sample is now cooling instead of warming. t = 95 s More magnetic domain walls form as magnetic anisotropy increases. These are visible as white and black vertical lines through center of field of view. Some pin at “C”-shaped dislocation. t = 103 s More magnetic domain walls growing in from bottom of field of view with monoclinic phase. t = 108 s Twin wall forms diagonally from bottom left toward middle right. Type 2 twins can also now be seen spreading from middle of field of view to the left (having already filled right side of sample). Their leading edge is a white domain wall. t = 109 s White magnetic domain wall/twin boundary continues to sweep left across top of field of view, briefly pinning at some dislocations. Type 2 twins have formed in the region right of this domain wall. t = 110 s White magnetic domain wall/twin boundary pins briefly at dislocation “C” in upper left corner. Then unpins and continues sweeping left. t = 112 s White magnetic domain walls/twin boundary forms type 1 twin wall as it meets a different region of type 2 twins in the upper left corner.
